# Recent Advances in the Neuropsychology of Pedophilia

**DOI:** 10.5964/sotrap.10515

**Published:** 2023-11-06

**Authors:** Shelby D. Leverett, Gilian Tenbergen

**Affiliations:** 1 Division of Biology and Biomedical Sciences, Washington University in St. Louis, St. Louis, MO, USA; 2 Department of Psychology, State University of New York at Oswego, Oswego, NY, USA; Department of Psychiatry and Psychotherapy, University Medical Center Mainz, Mainz, Germany

**Keywords:** pedophilia, neuropsychology, sexual abuse, minor attraction, executive functions

## Abstract

A considerable amount of research has investigated the relationship between potential neuropsychological dysfunction, pedophilia, and sexual offending against children. Until recently, these studies focused primarily on the relationship between executive functions and sexual offending against children, collapsing across underlying sexual preferences, like pedophilia. Prior research suggests neuropsychological dysfunction in individuals who have committed child sexual abuse. However, there are still unanswered questions about how these impairments relate to pedophilia as a sexual preference and whether these impairments are also observed in pedophilic individuals who do not offend. This review will discuss current findings as they relate to pedophilia, while using theoretical frameworks to guide future research.

## Highlights

- Pedophilia as a sexual preference is not synonymous with committing child sexual abuse. It is imperative that future studies do not conflate the two.
- Previously observed differences in neuropsychological function are driven by offense status (e.g., whether someone has committed child sex abuse), not by sexual preference.
- Observed differences occur mostly for the neuropsychological construct of impulsivity, specifically response inhibition.
- Future studies must include groups of nonoffending individuals with pedophilia to effectively differentiate the neuropsychological findings of illegal behavior from sexual preference.

## Abstract (plain language summary)

**Background:**

This review provides an in-depth discussion about our understanding of the role that neuropsychology plays in how child sexual abuse occurs and for whom.

**Why Was This Study Done?:**

Previous research has shown that engaging in child sexual abuse is related to impairments in executive functioning, specifically deficits in response inhibition. However, whether and how these impairments relate to sexual preferences, such as pedophilia, is still unclear.

**What Did the Researchers Do and Find?:**

Studies are urgently needed that specifically investigate these neuropsychological functions according to theoretical models which include groups of nonoffending pedophilic individuals.

**What Do These Findings Mean?:**

Future research should focus on recruiting and examining executive functions in this group to effectively differentiate the effect of sexual preference from that of the behavioral disorder.

Pedophilia is a paraphilia characterized by the ongoing sexual preference toward prepubertal children (Seto, 2008, 2018). Although the term *pedophilia* is often used synonymously with child sexual abuse, pedophilia itself refers only to the sexual preference (Tenbergen et al., 2015). According to the ICD-11 and the DSM-5, this paraphilia can be differentiated into two categories: *Pedophilia* and *Pedophilic Disorder* (APA, 2013; 11th ed., ICD-11; World Health Organization [WHO], 2019).

Pedophilia describes the sexual preference for prepubescent children – in the absence of either acting upon that preference in any way, or experiencing distress or impairment related to the preference. Pedophilic disorder, on the other hand, requires that the individual reports that A) the urges and fantasies are distressing or life-impairing, B) that they have acted upon these urges by sexually abusing a child or by consuming child sexual abuse material (CSAM), or C) they have experienced both symptoms (distress and behavior). These symptoms must be present for at least six months and be primarily directed toward prepubescent children. Thus, *pedophilia* describes a sexual preference to prepubescent children, while *pedophilic disorder* is a mental health diagnosis. A pedophilic individual can have a gender preference (e.g., heterosexual, homosexual, or bisexual pedophilia), can be exclusively attracted to children, or can also be attracted to individuals in other age groups (e.g., exclusive or nonexclusive pedophilia, respectively), and can be attracted to children within or outside of the family unit (APA, 2013).

It is imperative to note that pedophilia and child sexual abuse (CSA) are not synonymous. There are individuals with pedophilia who live law-abiding lives, without ever engaging in child sexual abuse behaviors (contact CSA or online CSAM). Conversely, there are individuals who do not have a sexual interest in children who go on to commit CSA offenses. As we discuss in this paper, distinctions must be made between the behavior of an individual (i.e., engaging in CSA), and the sexual preferences of that individual (i.e., pedophilic or nonpedophilic). Recent estimates suggest that only 25-50% of individuals incarcerated for CSA offenses are actually pedophilic, for the majority of cases involve offenders without sexual attractions to children (Beier et al., 2015; Schaefer et al., 2010; Seto, Cantor, & Blanchard, 2006). The historical reliance on behavior as a proxy for sexual preference (e.g., using CSA offenses as an indication of pedophilia) has led to a number of imprecise conclusions about the nature of pedophilia.

A related term, *Minor Attracted Person (MAP),* has emerged both in the scientific literature and in broader societal discourses (Jahnke et al., 2022; B4U-ACT, 2020). This term has social value, as it is less heavily stigmatized than the term pedophile, however it is neither a scientific nor a clinical term. Therefore, for ease of understanding, we will use the term pedophilia throughout this paper to refer to a sexual preference specific to prepubescent children.

## Neuropsychology of Pedophilia and Pedophilic Disorder

A considerable amount of research has investigated the relationship between potential neuropsychological deficits, pedophilia, and sexual offending against children. This paper will review neuropsychological findings in pedophilia while accounting for offense status. Additional discussions can be found in Turner and Rettenberger (2020) and Dillien et al. (2020), both of whom provided in-depth discussions about the role of neuropsychology in child sexual abuse. Finally, we will use theoretical frameworks to guide a discussion about directions for future research.

### Early Studies: Neuropsychological Findings in Sexual Offenders

Over the last 20 years, many studies have investigated the relationship between neuropsychological function and child sexual abuse behavior. However, these studies should be interpreted cautiously. Early studies (e.g., Flor-Henry, 1987) pooled multiple types of individuals with offense histories (i.e., child sex offender, adult offender, violent offenders, etc.) into the same group, and made comparisons with healthy controls. Not only did this pooling potentially obscure any differences that did exist, but it also prescribes that any results can only be interpreted in the context of general offending behavior without any specificity for type of offense or sexual preferences. Overall, these studies yielded mixed results; some studies found neuropsychological deficits in sex offenders relative to healthy controls (e.g., Hucker et al., 1988; Hucker et al., 1986) that others were unable to replicate (Abracen et al., 1991; Gillespie & McKenzie, 2000; Tarter et al., 1983).

More recently, studies have deliberately sought to investigate neuropsychological functioning in homogenous subgroups of individuals who have committed sexual offenses. Among the first studies to find meaningful executive function differences, Joyal et al. (2007) investigated neuropsychological functioning among individuals who sexually offended against children, individuals who offended sexually against adults, and demographically-matched normed data. Both offense groups (child and adult) performed normally (and similarly) on measures of cognitive flexibility, set shifting, and visual-spatial integration. However, relative to norms, both offense groups exhibited deficits in verbal fluency and verbal learning. Interestingly, individuals who offended sexually against children exhibited deficits in response inhibition that were not present in those with sexual offenses against adults. As behavioral inhibition can be correlated with impulsivity, subsequent studies directly assessed whether pedophilia and/or child sexual abuse (CSA) are associated with impulsivity. This was the case with a later study by Turner et al. (2018) who investigated impulsivity in the context of sexually relevant cues. They again found that relative to healthy nonoffending controls, individuals who offended sexually against children displayed deficits in response inhibition and behavioral impulsivity.

One limitation of these studies is that their methodologies limit the ability to draw conclusions about *pedophilic* populations. Many studies used a group of individuals with sexual offenses against children (defined as committing and/or admitting to at least one sexual offense against a prepubescent child) as a proxy for pedophilic interest, without directly assessing pedophilic interest. However, research has reliably demonstrated that individuals commit child sex abuse for many reasons, exclusive of pedophilic interest, such as antisociality, substance abuse, lack of age-appropriate peers, alleviating negative emotional states, etc., (Mann & Hollin, 2007; Seto, 2008, 2019; Tenbergen et al., 2015). So, to better understand pedophilia, studies shifted to examining individuals with child sexual offense histories who had also been diagnosed with pedophilia. While still less than ideal (a more ideal scenario would include assessing pedophilic individuals with no history of offense or incarceration), pedophilic offenders represent a population that is more visible and easier to access (e.g., from psychiatric or criminal referral centers, etc.).

### Neuropsychological Findings in Pedophilic Populations

In this vein, the following studies assessed pedophilic interest more directly and in ways that are consistent with best practices in the field; via self-report of a primary sexual attraction to prepubescent children (Eastvold et al., 2011; Massau et al., 2017; Suchy et al., 2009), a DSM-IV diagnosis of pedophilia (Cohen et al., 2002; Kruger & Schiffer, 2011; Schiffer & Vonlaufen, 2011), a penile plethysmography revealing greater arousal to prepubescent children than to adults (Eastvold et al., 2011; Suchy et al., 2009), or with the well-validated assessment, The Screening Scale for Pedophilic Interest (SSPI) (Seto & Lalumière 2001; Seto et al., 2004; Suchy et al., 2009).

#### Pedophilic Offenders vs Healthy Controls

Cohen et al. (2002) investigated a sample of male, heterosexual, nonexclusive pedophilic individuals who offended sexually against children relative to healthy controls and found no significant neuropsychological differences in set shifting, attention, impulsivity or verbal fluency.

Kruger and Schiffer (2011) investigated incarcerated individuals with pedophilia exclusively attracted to prepubescent children with a history of contact sexual offenses against at least two victims. After accounting for important covariates (i.e., age and education), analyses demonstrated that the pedophilic offender group noted more errors in cognitive flexibility and abstract reasoning than healthy controls. While these results suggest executive functioning weaknesses in the pedophilic group, the absolute values were within the normal range for all tasks. These results suggest that individuals with pedophilia who sexually offend against children are not driven by executive functioning impairments specifically, but rather slight weaknesses in various domains.

#### Pedophilic Offenders vs Nonpedophilic Offenders

As phenomenological and psychological research began to demonstrate differences between pedophilic and nonpedophilic individuals with sexual offense histories against children (i.e., distinct reasons for offending) (Seto, 2009, 2018, 2019), researchers began to focus on neuropsychological differences *between* these groups.

In what may have been the first study of this class, Suchy et al. (2009) investigated differences in neuropsychological profiles across groups of pedophilic individuals who offended sexually against children, nonpedophilic individuals who offended sexually against children, and healthy nonoffending controls. While no group differences were found in auditory memory, visual memory, or motor speed, both child offender groups exhibited weaknesses in executive functioning (a composite of response inhibition, cognitive flexibility, and working memory tasks). This is consistent with other reports of executive functioning deficits in populations of individuals with sexual offenses against children (i.e., Joyal et al., 2007; Turner et al., 2018). However, in comparisons of pedophilic offense and nonpedophilic offense groups, pedophilic individuals with child sexual offense histories displayed weaknesses in processing speeds relative to both nonpedophilic individuals with sexual offenses against children and healthy controls. Interestingly, this finding is inconsistent with previous assumptions (e.g., Flor-Henry, 1987) and findings of greater impulsivity in child sex offenders (e.g., Joyal et al., 2007). Instead, the authors posit that slower processing speeds may suggest a more deliberate response style, reflecting greater self-monitoring required to conceal sexual attraction to children in everyday life.

In a follow up study, Eastvold et al. (2011) found no evidence of generalized executive functioning *deficits* among three groups of pedophilic individuals who offended sexually against children, nonpedophilic individuals who offended sexually against children, and individuals with nonsexual criminal offense histories. Instead, each group showed distinct neuropsychological profiles, or pattern of *weaknesses*; such that, group scores for specific aspects of executive function significantly differed from each other, even though most scores were within normative ranges. Relative to the nonpedophilic group and non-sexual group, the pedophilic group demonstrated poorer behavioral inhibition. Interestingly, this relative weakness in inhibition was driven by slower response times in the pedophilic group, a finding that is consistent with that of Suchy et al. (2009). However, when considering inhibition accuracy, the pedophilic group was more accurate than the nonpedophilic group. Pedophilic individuals who had offended sexually against children also demonstrated better abstract reasoning and planning abilities relative to the nonpedophilic and non-sexual offense groups. The authors interpreted these results as neuropsychological support for grooming behavior (e.g., planning access to a victim without being apprehended), which could indicate a planning-oriented response style (Eastvold et al., 2011). More recent research does not appear to support this hypothesis (Massau et al., 2017).

Finally, Schiffer and Vonlaufen (2011) found that relative to individuals with nonsexual offenses and healthy controls, child sex offense groups (both pedophilic nonpedophilic) indeed exhibited deficits in response inhibition- consistent with earlier reports. Interestingly, the nonpedophilic sexual offense group also exhibited global executive function impairments that were not seen in a group of pedophilic sexual offenses. Specifically, nonpedophilic individuals displayed deficits in set-shifting, verbal memory, and response inhibition.

In a push to separate the effects of offense behavior from sexual preference, Massau et al. (2017) compared executive function across groups of child sex offenses with and without pedophilia. Interestingly, child sexual offending seemed to relate to deficits in response inhibition (consistent with findings in Turner et al., 2018 and Joyal et al., 2007), while pedophilia was more closely associated with reflection impulsivity (e.g., jumping to conclusions more quickly). It is important to note, however, that these effects did not survive multiple comparison corrections.

When taken together, these findings support a main effect of child sexual abuse, whereby child sexual abuse is associated with impulsivity. These findings provide interesting nuance, wherein although individuals with pedophilia who offend sexually against children tend to have slower responses than nonpedophilic individuals, the nonpedophilic individuals tend to exhibit more pervasive neuropsychological impairments. While these studies make important contributions to the field, it is important to note that without a control group of nonoffending pedophiles, it is difficult to conclude whether any results are associated with pedophilia itself, with the act of committing child sexual abuse, or with any of the confounds of general criminality or incarceration (Cantor & McPhail, 2016; Turner & Rettenberger, 2020).

Future studies should also carefully control for psychiatric and neurological comorbidities, incarceration status, and offender status, as these are frequent confounds in the reviewed studies. Few studies to date have examined neuropsychological deficits in these subsamples, but these findings would help understand whether these confounds are mediating factors in the pathway to offense behavior or whether they are related to the pedophilic sexual preference (Cantor & McPhail, 2016; Turner et al., 2018; Turner et al., 2020; Turner & Rettenberger, 2020).

### Contact Sexual Offenses vs Noncontact Sexual Offenses

Emerging studies are not only investigating neuropsychological function relative to whether an individual with pedophilia has committed an offense or not, but also have started to investigate neuropsychological function relative to different types of sexual offenses against children (e.g., contact vs noncontact offenses). For further review, please see Dillien et al. (2020). Contact sexual offenses involve the presence of a physical victim, while noncontact sexual offenses refer to internet offenses (e.g., viewing and distributing child sexual abuse material (CSAM), online solicitation offenses, etc.). Offense type is an important factor to consider, as research has elucidated differences between these two groups that potentially suggest two different pathways to offending. First, relative to contact offense groups, noncontact offense groups are typically younger, more highly educated, more intelligent, and possess fewer antisocial traits and lower rates of criminality (Babchishin et al., 2011; Babchishin et al., 2018; Blanchard et al., 2007; Neutze et al., 2011). Importantly, contact and noncontact offense groups also differ in their levels of pedophilic interest, whereby individuals with noncontact offenses exhibit higher levels of pedophilic interests than those with contact offenses (Seto et al., 2006; Seto et al., 2017).

Babchishin et al. (2011) postulated that individuals with noncontact sexual offenses have greater rates of self-control and are less impulsive than comparable individuals with contact offenses. This explanation is still under investigation, with previous studies providing equivocal results (some suggest lowered rates of impulsivity, Elliott et al., 2009, whereas others find no differences between offense types, Howitt & Sheldon, 2007). Interestingly, it appears that the transition from noncontact to contact offending is very low (estimates range from 2.0% – 12%), which could suggest some inhibitory mechanisms are present and functional (Babchishin et al., 2022; Seto, 2018; Seto et al., 2011).

### Undetected Offenders and Pedophilic Nonoffenders

There are two groups that have not been discussed but are pivotal in our understanding of the relationship between neuropsychological function and pedophilia: pedophilic individuals who do not engage in any form of child sexual abuse (termed pedophilic nonoffenders) and pedophilic individuals who have engaged in child sexual abuse but are unknown to the justice system (termed darkfield offenders). To date, all investigations into the executive functioning of pedophilia have investigated pedophilic offenders (pedophilic sexual preference confounded with criminality and incarceration effects), nonpedophilic offenders (criminality and incarceration effects without a sexual preference), and nonoffenders (neither criminality/incarceration effects nor a sexual preference). However, to truly differentiate the neuropsychological effects of incarceration or general criminality from those of pedophilia as a sexual preference, the field urgently needs to recruit community-dwelling, non-incarcerated darkfield individuals with sexual offenses against children and pedophilic nonoffense groups. The double dissociation provided by including these two groups is the way to do just that.

## Threats to Validity

There are several crucial issues with validity in the literature, and while many of these threats were mentioned in the text, they merit additional attention. These threats fall under two main categories – internal and external validity.

### Internal Threats

Currently, the field has – at best – imperfect and imprecise tools to measure sexual interest and preference. These tools range from simple self-report to phallometric assessment using sexual stimuli. While there is not length in this paper to fully debate the measurement issues with sexual preference assessment, it is worth stating that a threat to internal validity in these studies is the inclusion of groups whose pedophilic sexual preferences were assessed using tools that may or may not be entirely accurate. These tools are certainly an improvement over the historical trend of using behavior as a proxy for interest, but are still limited in their interpretability.

Additionally, there are threats related to how we both define and measure executive function. Executive function is a complex set of neuropsychological cognitive processes that are not easily measured. Some standing criticisms of studies of executive function include: the reliance on tests that are not standardized or that do not sufficiently separate executive function from other cognitive processes, inconsistently operationalizing executive function across studies, and the use of imprecise and varied assessment methods (Diamond, 2013; Suchy et al., 2009). All represent considerations that scholars should address in future studies when investigating the neuropsychological – and the executive functioning of – individuals with and without histories of sexual offenses against children.

Finally, a threat to internal validity arises with the use of convenience samples who may have multiple comorbid psychiatric diagnoses and/or criminological histories that confound the relationship among neuropsychological dysfunction, sexual offending behavior, and sexual preference. Future studies should address these issues when recruiting and testing their samples.

### External Threats

A primary concern for future scholars would be the use of appropriate control groups. The use of adequate control groups impacts a study’s external validity by way of reducing result generalizability across studies. Future research should carefully consider the outcome variables and how to compose appropriate control groups that avoid systematic confounds.

Finally, a major threat to external validity that has plagued the field for some time is the use of highly self-selected samples volunteering for research. The role of self-selection in sex research is a known bias (see Dunne et al., 1997; Fenton et al., 2001) and is a particularly salient topic for studies where incarcerated samples are used. Future research should be aware of this self-selection bias and where possible, should use normative or community samples to provide necessary metrics for assessing populations with presumed deficits or impairments.

## Theoretical Implications

The dearth of literature that specifically investigates darkfield offense groups and pedophilic nonoffending individuals provides a ripe field for investigation. Not only will it better our understanding of a variety processes in pedophilia (i.e., behavioral, neural, psychiatric, phenomenological, etc.), but it also has the potential to provide insight for ongoing crucial questions in the field, such as whether pedophilia has neurodevelopmental origins like those of *autism spectrum disorders* (ASD) or schizophrenia, as debated by Fazio (2018) and Joyal et al. (2019).

Further, this double dissociation could contribute to the development of explanatory theories about the origin of pedophilia, such as Gannon’s recent paper on the *Compositional Explanatory Theory of Pedophilia (CEToP)* (Gannon, 2021). In an effort to reduce methodological heterogeneity in the operationalization of pedophilia and to rectify inconsistent etiological findings, Gannon provides an etiological framework for different presentations of pedophilia. Reconciling seemingly conflicting evidence from biological (e.g., prenatal, neurodevelopmental, epigenetic) and environmental (e.g., social learning) explanations of pedophilia, the theory posits that differential contributions from each of these pathways result in either a less severe (less exclusive) pedophilic sexual *interest* or a more stable, pervasive pedophilic sexual *preference*. Perhaps neuropsychological findings from nonoffending pedophilic individuals could provide insight into these two attractions and their etiologies, while eliminating some of the confounds that are pervasive in the literature.

Finally, investigations into neuropsychology of nonoffending pedophilic individuals could also inform leading explanatory theories about the onset of sexual offending behavior. The Motivation-Facilitation Model (MFM) posits that a combination of three factors – motivational, facilitation, and state – work together to either enable or inhibit sexual offending behavior (Seto, 2019). Neuropsychological findings in nonoffending pedophilic individuals could elucidate potential protective factors that actually prevent offending. For example, can we identify neuropsychological functions that act as trait factors to decrease facilitation? This would be especially relevant in instances where individuals have the same motivation (e.g., pedophilia), but differ (or hope to differ) in offense behavior. Could that neuropsychological function then be used as a tangible target for intervention or prevention? By incorporating nonoffending pedophilic populations, the MFM model can serve as an adept guide for future research into the nuance of sexual offending behavior.

Scholarship in this field will be invaluable in gaining a holistic, multidimensional understanding of pedophilia as a sexual preference, of the nature of sexual offending behavior, and perhaps most critically, of the distinction between the two.

## Conclusions and Future Directions

As we have seen, the most pronounced neuropsychological impairments appear to apply to individuals who have committed contact child sexual abuse, and primarily for individuals *without* pedophilia. The critical point here is that these are individuals who have contact offenses but who do not have pedophilia – which suggest a stronger neuropsychological role in the behavioral regard (i.e., CSA). However, it remains to be seen exactly how (or whether) these impairments relate to pedophilia as a sexual preference. Addressing this gap will not only deepen our understanding of pedophilic preference, but also deepen our understanding of the ways in which *pedophilic preference* is distinct from *pedophilic disorder*. This could be tangibly consequential both for the field and for society at large. This could provide critical guidance in differentiating between offense trajectories among those with pedophilic sexual preference. For example, is there a set of neuropsychological markers that are correlated specifically with nonoffending in pedophilia? Is there a set of neuropsychological markers that can explain – and hopefully predict – whether any of those nonoffending individuals with pedophilia might transition to pedophilic offenders? In this way, we could also begin to understand the development of *pedophilic disorder*. If this question were to be systematically addressed in the literature, the results could immediately translate to clinical prevention efforts and to supportive work with various subpopulations of pedophilic individuals.

The field has made a lot of progress in the last decade about the nature of neuropsychological functioning between different types of sexual offending in individuals with and without pedophilia. Response inhibition and employing ecologically valid designs with diverse subsamples of pedophilic individuals (e.g., nonoffending pedophilic individuals) will continue to be a direct target for scholars wishing to understand the role of general criminality and neuropsychological differences in pedophilia. It will be very exciting to see a new era develop in pedophilia research that seeks to understand not only the relationship between pedophilia and behavior, but also better understand pedophilia itself.
